# Rice Hull-Derived Carbon for Supercapacitors: Towards Sustainable Silicon-Carbon Supercapacitors

**DOI:** 10.3390/polym13244463

**Published:** 2021-12-20

**Authors:** Changwei Li, Honglei Chen, Liqiong Zhang, Shenghui Jiao, Huixin Zhang, Junliu Zhang, Peng Li, Yubo Tao, Xin Zhao

**Affiliations:** 1 State Key Laboratory of Biobased Material & Green Papermaking, Faculty of Light Industry, Qilu University of Technology, Shandong Academy of Sciences, Jinan 250353, China; lichangwei0205@126.com (C.L.); chenhonglei_1982@163.com (H.C.); zhang765882@163.com (L.Z.); 13783261144@163.com (S.J.); zhanghuixin2021@163.com (H.Z.); zjl17860657187@163.com (J.Z.); lipeng@qlu.edu.cn (P.L.); taoyubo@qlu.edu.cn (Y.T.); 2 Key Lab of Biomass Energy and Material, Jiangsu Province, Jiangsu Co-Innovation Center of Efficient Processing and Utilization of Forest Resources, Institute of Chemical Industry of Forest Products, Chinese Academy of Forestry, Nanjing 210042, China

**Keywords:** rice hull, Si-carbon material, supercapacitor, carbonization-activation

## Abstract

A simple and effective mixing carbonization-activation process was developed to prepare rice hull-derived porous Si–carbon materials. The morphologies and pore structures of the materials were controlled effectively without any loading or additions at various carbonization temperatures. The structures of the samples changed from large pores and thick walls after 800 °C carbonization to small pores and thin walls after 1000 °C carbonization. An additional alkali activation–carbonization process led to coral reef-like structures surrounded by squama in the sample that underwent 900 °C carbonization (Act-RH-900). This optimal material (Act-RH-900) had a large specific surface area (768 m^2^ g^−1^), relatively stable specific capacitance (150.8 F g^−1^), high energy density (31.9 Wh kg^−1^), and high-power density (309.2 w kg^−1^) at a current density of 0.5 A g^−1^ in 1 M KOH electrolyte, as well as a good rate performance and high stability (capacitance retention > 87.88% after 5000 cycles). The results indicated that Act-RH-900 is a promising candidate for capacitive applications. This work overcomes the restrictions imposed by the complex internal structure of biomass, implements a simple reaction environment, and broadens the potential applicability of biomass waste in the field of supercapacitors.

## 1. Introduction

Porous structures have existed in nature for a very long time, for example, in charcoal, biological tissues, and rocks. Based on the IUPAC classification, porous materials can be divided, based on their pore diameters, into microporous (<2 nm), mesoporous (2–50 nm), and macroporous (>50 nm) materials [1]. Hierarchically porous materials have attracted much attention and have been widely used in sustainable development fields, including energy storage, life science, gas separation, nanoscience, catalysis, and sensors [2]. One key aspect that has emerged from the worldwide promotion of sustainable development strategies is the implementation of renewable resources derived from the effective utilization of neglected biomass. Rice is one of the most important food crops in China, and it accounts for about half of the country’s total grain output. Rice hull is one of the main byproducts of rice processing, corresponding to about 20 wt.% of the rice quantity. However, rice hull is generally used as feed stuffing for livestock and bedding for livestock stables; thus, it is not valorized for other applications. The neglected rice hull waste biomass is composed of cellulose, hemicellulose, lignin, and more than 20% silicon [3]. The chemical composition and biological structure of rice hull are suitable for the preparation of biomass-based carbon materials [4]. Biomass-derived carbon substances represent promising supercapacitor materials owing to their renewable nature, the low cost of the original resources, their abundantly porous structural features, and their controllable pore size distribution [5].

Recently, rice hull carbons have been prepared by one–step steam activation, but the surface areas of these materials are relatively lower (197 and 273 m^2^ g^−1^) [6,7]. This is attributed to the fact that silicon may jam the pore structure, resulting in inhomogeneous pore size distribution, which further affects the ion transport channel and storage space, restricting the transformation of rice hull into biomass functional materials. Therefore, the silicon in rice hull is generally used as a template and then removed by an alkaline etching process in a one- or two-step method to prepare biomass-based carbon materials with high porosity [8]. Rice hull-based carbon materials with a low ash content and high specific surface area were obtained by using hydrofluoric acid and continuous pyrolysis to desiliconize and activate rice hulls [9], and the desiliconized rice hull–based carbon material was prepared by using 6 M NaOH to dissolve silicon into Na_2_SiO_3_ [10]. These approaches do not allow for the full utilization of the silicon source. Moreover, the preparation of silicon-carbon materials is most commonly achieved by introducing an external silicon source [11]. Only a limited number of reports describing the preparation of biomass-derived silicon-carbon composites employ native silicon or discuss energy-storage applications [12]. Rice hulls used as silicon-fixing biomass are mainly composed of cellulose (~33%), hemicellulose (~26%), lignin (~7%), and amorphous silicon dioxide (~20%) [13]. Therefore, it is crucial to develop methods that enable full exploitation of the inherent advantages of the biomass resources. Specifically, by relying on the internal relationship between silicon and carbon in the rice hull, it is necessary to design a simple and effective process to obtain silicon-carbon composites with controllable energy-storage properties from silicon-fixing biomass [14]. Using silicon-fixing biomass (i.e., rice hull) to prepare silicon-carbon materials with designed electrochemical and energy-storage properties widens the scope of efficient value-added conversion of agricultural waste resources, while also further expanding their potential use in supercapacitor development [15].

In this work, silicon-carbon materials were successfully obtained from silicon-fixing biomass (i.e., rice hull). The porous structures of the samples were controlled, and the electrochemical performances of the materials were optimized by coupling carbonization and alkali mixing carbonization processes. This work overcomes the restrictions imposed by the complex internal structure of the rice hull biomass, demonstrates high–efficiency utilization of silicon in silicon-rich biomass, and broadens the scope of silicon–rich waste in supercapacitor applications.

## 2. Experimental Section

### 2.1. Materials

**Figure 1:**
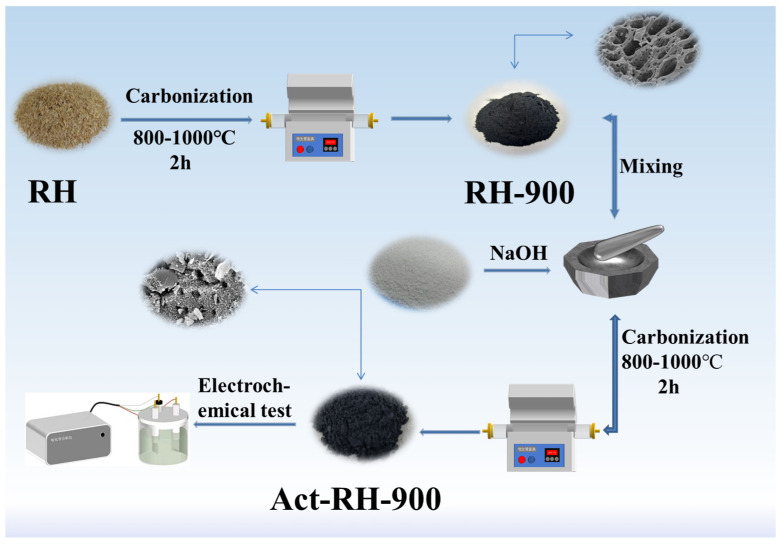
The schematic cartoon of samples.

The rice hulls were sieved (60 mesh, Lingshou-Hengchang company, Hengchang, China), washed with distilled water, dried at 105 °C, and then a certain amount of rice hull was placed in a crucible and placed in a tube furnace, carbonized at various temperatures (800, 900, or 1000 °C) for 2 h under N_2_ atmosphere at a heating rate of 5 °C min^−1^. The obtained rice hull-based carbon materials are herein denoted as RH-x, where x is the carbonization temperature. Later, the superior RH–900 sample was ground into a powder and mixed with NaOH powder in a 2:1 ratio. The resulting mixtures were further carbonized at 800, 900, or 1000 °C for 2 h under N_2_ atmosphere at a heating rate of 10 °C/min. The activated samples were washed first with 10 wt.% HCl and then with distilled water until they reached a neutral pH, and finally, they were dried at 105 °C for 6 h. The obtained activated RH-x samples are herein denoted as Act-RH-X, where X is the carbonization temperature applied after the activation of RH-900. The schematic cartoon is shown in Figure 1.

### 2.2. Characterization

The morphologies and pore textures of the obtained samples were investigated using scanning electron microscopy (SEM; JSM-7401F microscope, Hitachi, Tokyo, Japan) operating at an acceleration voltage of 10 kV. Transmission electron microscopy (TEM) images were obtained on a JEOL 2011 apparatus (JEOL, Hokkaido, Japan) operating at 200 kV. The surface composition and chemical state of the obtained samples were investigated by X-ray photoelectron spectroscopy (XPS; Escalab 250Xi, Thermo Fisher Scientific, Waltham, MA, USA) with an Al Kα X-ray source. Powder X-ray diffraction (XRD) patterns of the samples were measured using a Bruker D4 powder X-ray diffractometer (Bruker, Rheinstetten, Germany) with Cu Kα radiation at 40 kV and 40 mA. Raman spectroscopy (Renishaw inVia, London, UK) with a He-Ne laser source (λ = 532 nm) was used to analyze the texture of the biomass-based carbon composites. Nitrogen sorption isotherms were measured with a Micromeritics ASAP 2460 sorptometer (Maike, Georgia, USA) using nitrogen as the adsorbate at 77 K. All samples were degassed at 150 °C for more than 10 h prior to the analysis. The surface area (S_BET_) was calculated using the Brunauer–Emmett–Teller (BET) method based on the adsorption data in the relative pressure range of 0.02–0.35, and the total pore volume was determined at the highest relative pressure.

### 2.3. Electrochemical Performance Tests

Cyclic voltammetry (CV; at various scan rates and voltage ranges), galvanostatic charge/discharge tests (GCD), and electrochemical impedance spectroscopy (EIS; from 0.01 Hz to 10 kHz with a potential amplitude of 50 mV) were performed on 0.01 g samples using an electrochemical workstation (IVIUM, Netherlands). The tests were conducted at room temperature using a three-electrode system, in which mercury/mercury oxide (Hg/HgO) was used as the reference electrode, a platinum sheet was used as the counter electrode, and 1 mol L^−1^ KOH solution served as the electrolyte. The working electrode was prepared by loading a slurry comprising 80 wt.% active material (e.g., biomass-based carbon composites that had been ball-milled for 24 h), 10 wt.% conducting agent (i.e., carbon black), and 10 wt.% binder (i.e., polytetrafluoroethylene in methanol) onto nickel foam. The obtained samples were mixed with carbon black and polytetrafluoroethylene in a ratio of 8:1:1, and the appropriate amount of pyrrolidone solution was added to the mixture. In order to mix the sample uniformly, it was sonicated for 15 min. A small amount of the mixed liquid was sucked up by the syringe, and the liquid was evenly dropped on the nickel sheet. The nickel sheet needed to be dried at 105 °C for 12 h, which was extruded by a sheeter under a pressure of 6 MPa for 30 s. The specific capacitance and energy density were measured after three repeated GCD experiments to obtain the average value.

The capacitance of each sample was calculated from the discharge plots using Equations (1) and (2):

*Q* = *C* × *V* (1)

*Q* = *I* × *t* (2)

where *Q* is the stored charge (F g^−1^), *I* is the current (A), and *V* is the change in potential within the discharge time, *t* (s).

The energy density, *E* (Wh kg^−1^), and power density, *P* (W kg^−1^), of each sample were calculated from the discharge plots using Equations (3) and (4), respectively:

*E* = (*C* × *V*^2^)/2 (3)

*P* = *E*/*t* (4)

## 3. Results and Discussion

### 3.1. SEM

**Figure 2:**
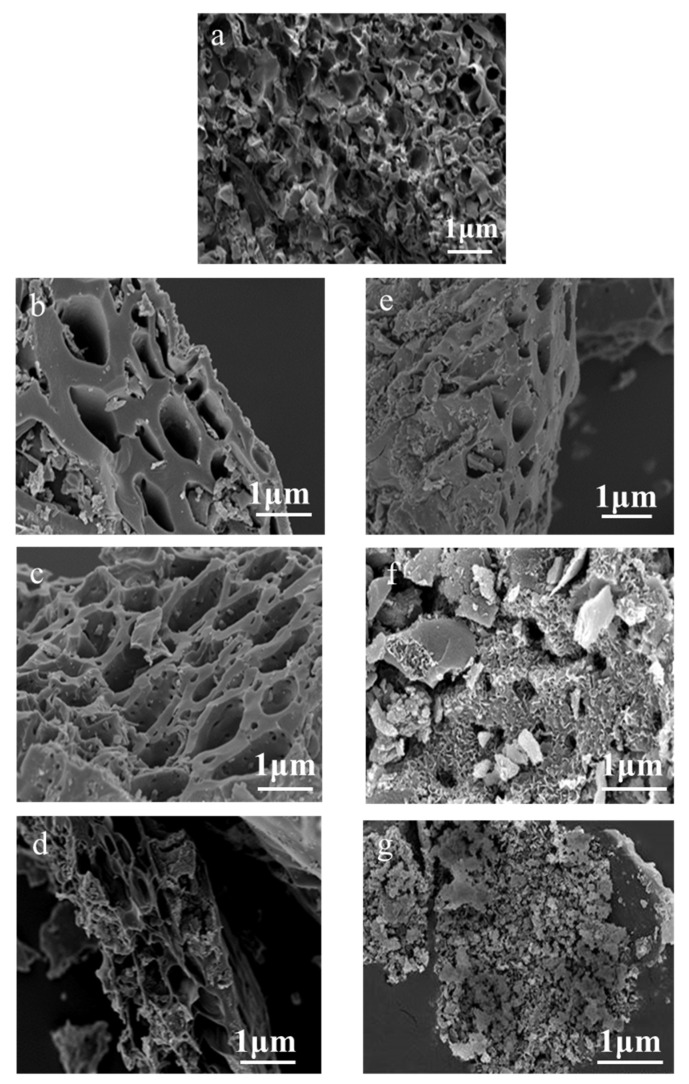
SEM images of: (**a**) RH, (**b**) RH-800, (**c**) RH-900, (**d**) RH-1000, (**e**) Act-RH-800, (**f**) Act-RH-900, and (**g**) Act-RH-1000.

The morphologies of the rice hull-based carbon samples were evaluated by SEM, and the obtained images are presented in Figure 2a–g. The untreated rice hull (i.e., RH; Figure 2a) contained a skeleton of interconnected holes derived from the natural structural features of rice hull. These characteristics are conducive to the formation of highly porous carbon materials with specialized structures. As shown in Figure 2b–d, the RH carbon materials obtained following carbonization at different temperatures displayed distinct structural characteristics. For example, RH-800 exhibited large pores (diameter = 1 μm, wall thickness = 200–500 nm; Figure 2b). After higher-temperature carbonization, the pore wall of RH-900 (Figure 2c) was reduced to 100–300 nm, and numerous small pores appeared in the thin walls, thus highlighting the significantly higher porosity of this sample. When the carbonization temperature increased to 1000 °C, the pore walls in RH-1000 became thinner or were destroyed (Figure 2d), likely because of the continued volatilization of organic substances at the ultra-high applied carbonization temperature. The collapse of these structures could affect the performance of the obtained samples. These results suggested that the abundant multistage pores of RH-900 could be favorable for the further development of pore structures [16]. Following the RH-900 activation process, the resulting samples’ structures underwent additional changes (Figure 2e–g). 

The Act-RH-800 sample (Figure 2e) still contained pores, but a small amount of squama was also visible. In contrast, Act-RH-900 (Figure 2f) displayed coral reef-like structures (without pores) surrounded by numerous squama structures. The emergence of this morphology was attributed to the effect of alkali etching [17]. The observed squama-like defect structure provides a large contact area and numerous active sites for transport ions, which enhances the electrochemical properties and increases the potential applicability of biomass carbon materials for transmission and energy storage [18]. However, the pore structure of Act-RH-1000 disappeared, thereby revealing amorphous powder structures (Figure 2g), which were formed following the polymerization of samples via activation-carbonization at higher temperatures [19]. These results suggested that an appropriate carbonization temperature could alter the pore structures of materials, and effective activation etching could expose more active sites to enhance the applicability of silicon-fixing biomass-derived carbon materials in the field of supercapacitors [20].

### 3.2. TEM

**Figure 3:**
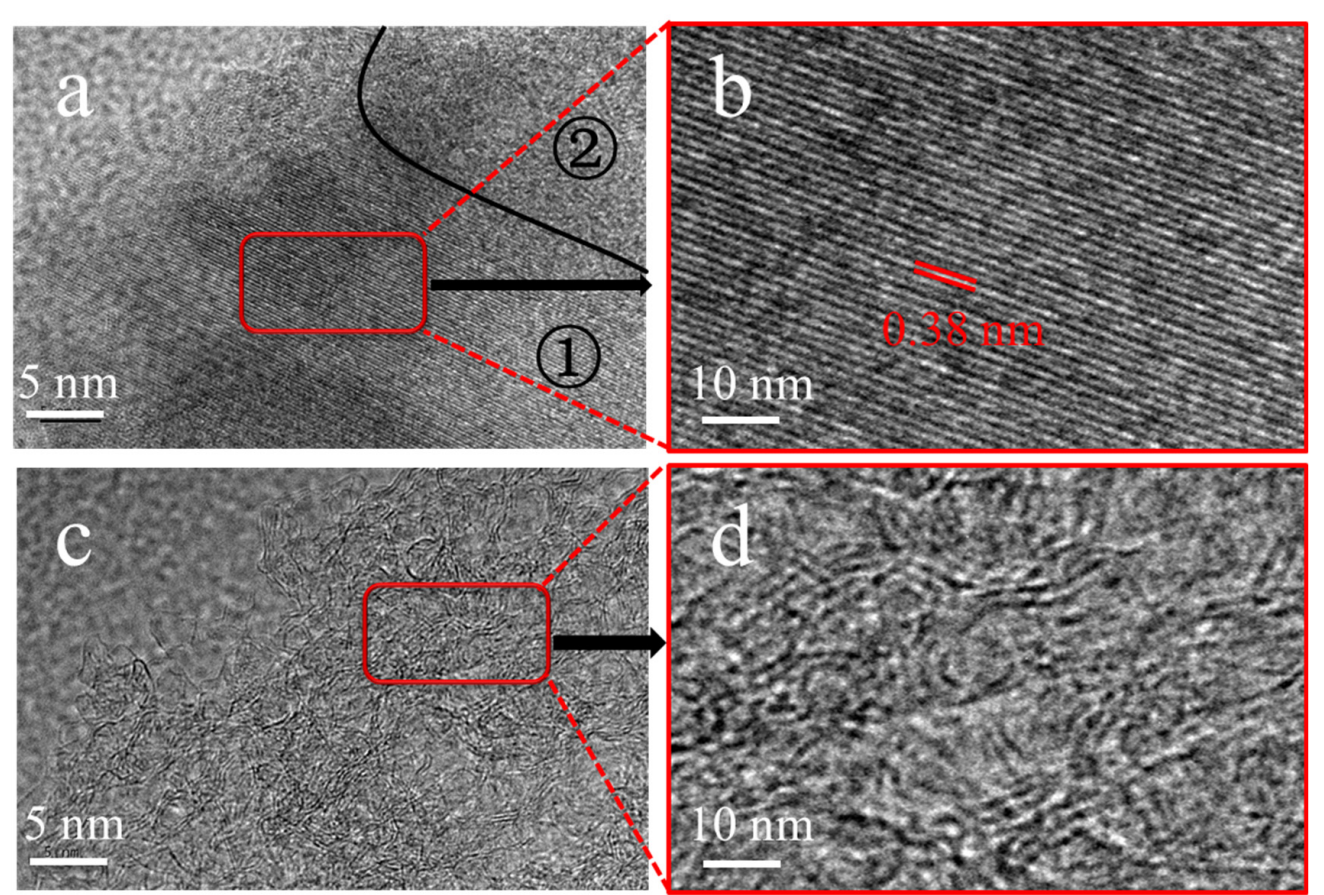
TEM images of (**a**,**b**) RH-900 and (**c**,**d**) Act-RH-900.

The pore textures of RH-900 and Act-RH-900 were further investigated by TEM, and the images are presented in Figure 2a–d. The RH-900 sample (Figure 3a,b) exhibited a two-dimensional ordered graphite lattice fringe in the region labeled (1) and several ordered structures with one-dimensional channels in the region labeled (2). These lattice fringes were formed by high-temperature carbonization, and such highly graphitized structures are beneficial for improving the rapid transmission of electrons [21]. However, portions of the ordered structures were destroyed in Act-RH-900 (Figure 3c,d), and the defective ordered structures generated by the etching effect of sodium hydroxide are beneficial for the formation of diverse pore sizes and pore structures [22]. Furthermore, certain defective structures with different pore sizes could provide more charge storage sites and more electrolyte ion adsorption sites, which would enhance the electrochemical performance of the carbon materials [23].

### 3.3. XRD and Raman

**Figure 4:**
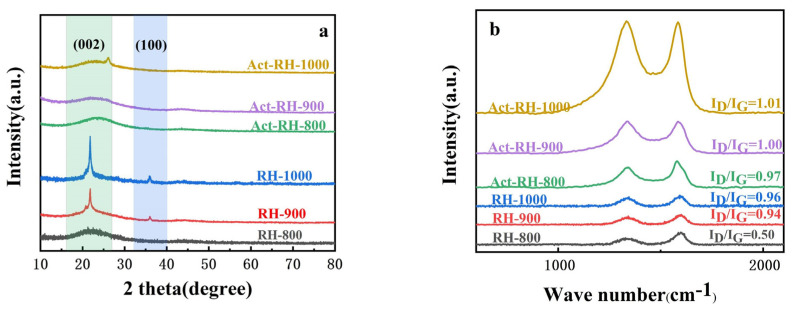
(**a**) XRD patterns and (**b**) Raman spectra of the investigated samples.

To better understand the crystal structures of the obtained samples, XRD experiments were carried out, and the results are shown in Figure 3. All of the obtained diffraction patterns have a carbon (002) peak in the range of 20–25° (Figure 4a), which confirms the amorphous characteristic of the carbon materials. In this region, RH-800 displays a small peak, but RH-900 and RH-1000 have sharp diffraction peaks, thus implying that higher carbonization temperatures lead to more graphitized structures [24]. In addition, the X-ray diffraction peaks around 20–25° for Act-RH-800, Act-RH-900, and Act-RH-1000 gradually broadened after the activation-carbonization process. This may have been a result of microscopic defects in the grain after the alkali activation or the fact that the X-rays were incident on a small crystal [25]. Meanwhile, a weak carbon peak (100) emerged around 35–40° in the RH-900 and RH-1000 XRD patterns, and this slight shift was attributed to some degree of ordered structures [26]. However, the peak (100) disappeared in the Act-RH-800, Act-RH-900, and Act-RH-100, indicating that activation-carbonization at higher temperatures could destroy the degree of order in carbon materials [27]. The defects, disorder, and graphitization in the samples were further investigated by Raman spectroscopy (Figure 4b). All of the samples had clear D and G peaks at approximately 1340 and 1580 cm^−1^, respectively. The D peak corresponds to disordered or defective carbon, and the G peak represents ordered carbon or the interplanar sp^2^ C–C stretching mode [28]. Furthermore, the degree of graphitization of the carbon materials could be measured based on the ratio of the relative intensities of the D band and the G band (*I_D_*/*I_G_*). The *I_D_*/*I_G_* values of RH-800, RH-900, and RH-1000 were 0.50, 0.94, and 0.96, respectively, and the *I_D_*/*I_G_* values of Act-RH-800, Act-RH-900, and Act-RH-1000 were 0.97, 1.00, and 1.01, respectively. The decreasing degree of graphitization was mainly attributed to the defective graphitization structures in the samples [29]. However, certain defective structures are beneficial because they provide more spaces for charge storage and more adsorption sites for electrolyte ions, which can enhance the electrochemical performance of carbon materials [30].

### 3.4. XPS

**Figure 5:**
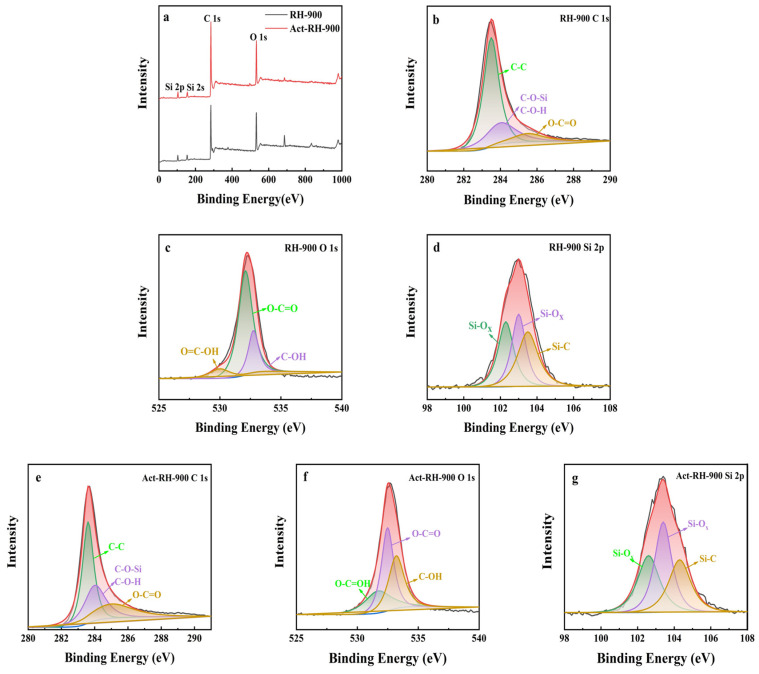
(**a**) XPS spectra and deconvoluted peaks for the (**b**–**d**) RH-900 and (**e**–**g**) Act-RH-900 samples.

The surface components of the samples were examined by XPS, which could reveal the changes in the electronic state of the primary skeleton, as well as variations in the texture and morphology of the samples following carbonization and alkali activation. The RH-900 and Act-RH-900 samples were primarily composed of C, O, and Si (Figure 5a). The C 1s peaks in the RH-900 spectrum could be deconvoluted to reveal three peaks at binding energies of 283.5, 284.0, and 285.6 eV (Figure 5b), which correspond to the C–C, C–O–Si/C–O–H, and O–C=O groups, respectively [31]. The O 1s core-level XPS spectrum (Figure 5c) of the RH-900 contains three Gaussian peaks that correspond to the O=C–OH, O–C=O, and C–OH moieties at binding energies of 530.0, 532.1, and 532.3 eV, respectively [32]. The Si 2p spectrum of the RH-900 sample was resolved into three Gaussian peaks (Figure 5d), corresponding to Si–O_x_ (102.2 and 103.1 eV) and Si–C (103.5 eV) groups [33]. The XPS spectra of Act-RH-900 were similar to the corresponding RH-900 spectra. The C 1s core-level XPS spectrum (Figure 5e) of Act-RH-900 contains three Gaussian peaks that correspond to the C–C, C–O–Si/C–O–H, and O–C=O moieties at binding energies of 282.8, 284.1, and 285.0 eV, respectively [34]. The O 1s peaks in the RH-900 spectrum could be deconvoluted to reveal three peaks at binding energies of 532.1, 532.5, and 533.1 eV (Figure 5f), which correspond to the O=C–OH, O–C=O, and C–OH groups, respectively [35]. The Si 2p spectrum of the Act-RH-900 sample was resolved into three Gaussian peaks (Figure 5g), corresponding to Si–O_x_ (102.5 and 103.5 eV) and Si–C (104.6 eV) groups [36]. On the basis of these results, the primary components of the samples were determined to be C–C, C–O, C–Si, and Si–O_x_ bonds. The stable carbon skeleton structure would be expected to improve the structural stability and the electrical conductivity of the materials [37]. The distinct elemental contents of RH-900 and Act-RH-900 are presented in Table 1: both samples had high carbon contents, which supported their stable structures. The Act-RH-900 sample contained higher oxygen (20.79 at.%) and silicon contents (8.12 at.%) than RH-900 (O: 17.59 at.%, Si: 6.7 at.%). Oxygen-containing functional groups and silicon active sites increase the wettability of carbon materials in aqueous electrolytes, and moreover, the results of these experiments indicated that silicon-containing functional groups in the biomass could significantly optimize the carbon skeleton by adjusting its morphology, structure, and electronic state. Ultimately, this improved the structural stability and conductivity of the samples, thus enhancing the pseudo-capacitance (i.e., the redox capacitance) of the resulting carbon-based supercapacitor materials [38]. 

### 3.5. BET

**Figure 6:**
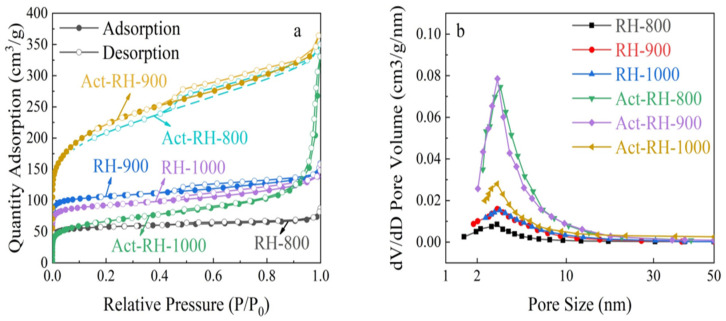
(**a**) Nitrogen adsorption-desorption isotherms and (**b**) pore size distributions of the investigated samples.

Figure 6 shows the nitrogen adsorption-desorption isotherms and the pore size distributions of the investigated samples under various conditions. The RH-800 sample presented a type-I isotherm, indicating significant microporous features, but the RH-900 and RH-1000 samples exhibited type-IV isotherms with H4-type hysteresis loops, indicating mesoporous features, which were attributed to the carbonization and polycondensation of aromatic compounds causing the volatilization of small molecules (CO_2_, CH_4_, CO, H_2_) during the carbonization process. However, RH-1000 had lower adsorption at a low relative pressure (P/P_0_ = 0.1) because of the partial collapse of the microporous structure [39]. The RH-900 sample had an excellent micropore to mesopore ratio, which is beneficial for charge transfer and storage during the charge and discharge processes [40]. The samples obtained after further alkali activation generally exhibited higher adsorption capacities. The strong adsorption observed at a low relative pressure (P/P_0_ = 0.1) and hysteresis loops in the P/P_0_ range of 0.45–1.0 nm indicated the presence of micropores and mesopores in the Act-RH-800 and Act-RH-900, respectively [41]. However, the similar type-IV isotherms confirmed their similar pore structure properties. In contrast, a tail was observed in the isotherm of the Act-RH-1000 sample in the relative pressure range of P/P_0_ > 0.9, which indicated the presence of macropores that formed following the collapse of the pore structure at a higher temperature (1000 °C) after alkali activation [42]. These results suggested that the porous structures of Act-RH-800 and Act-RH-900 have certain advantages in terms of charge transmission [43]. Therefore, the pore size distributions of the investigated samples under various conditions were evaluated (Figure 6b). In general, the pore structure was distributed within the range 2.0–5.0 nm (mainly concentrated around 3.0 nm). This hierarchical pore structure is very beneficial for enhancing the electrochemical performance in supercapacitors [44]. Pore diameters in the range of 2.0–5.0 nm could provide a suitable pathway for solvation ions, as well as storage space for electrochemical interactions/reactions. 

The pore structure parameters of the samples are listed in Table 2. The S_BET_ increased from 226 m^2^ g^−1^ (RH-800) to 418 m^2^ g^−1^ (RH-900) and then decreased to 286 m^2^ g^−1^ (RH-1000) as the carbonization temperature increased, and the S_micro_ reached 389 m^2^ g^−1^ corresponding to a pore volume of 0.246 cm^3^ g^−1^. After further alkali activation treatment, the S_BET_ was significantly improved: the S_BET_ increased from 732 m^2^ g^−1^ (Act-RH-800) to 768 m^2^ g^−1^ (Act-RH-900) and then decreased to 229 m^2^ g^−1^ (Act-RH-1000) as the carbonization temperature increased, and the S_micro_ reached 637 m^2^ g^−1^ corresponding to a pore volume of 0.563 cm^3^ g^−1^. In fact, the energy-storage capacity of functional electrochemical materials mainly depends on a high specific surface area and an appropriate pore size distribution [45]. The pore size distribution characteristics of Act-RH-900 provide significant storage space for ion transformation and transmittance, and decrease the impedance of electronic transport, which is advantageous for supercapacitor electrodes [46].

### 3.6. Electrochemical Properties

**Figure 7:**
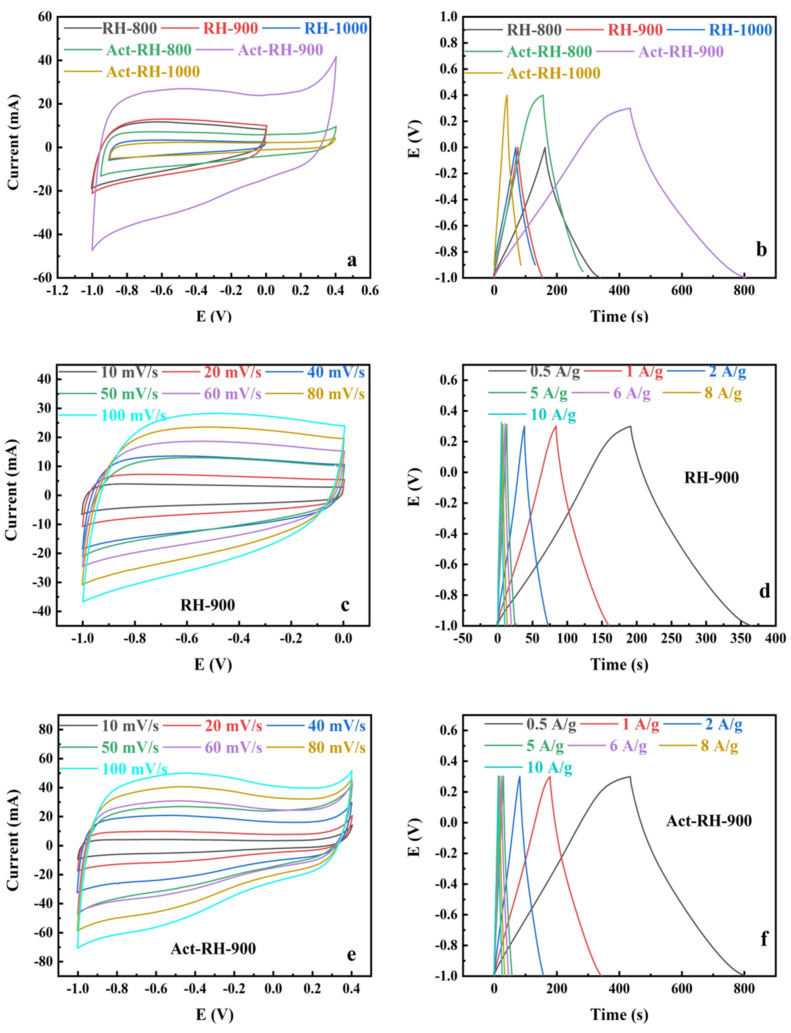
(**a**) CVs of all samples at a scan rate of 50 mV s^−1^. (**b**) GCD results for all samples. (**c**) CVs of RH-900 at scan rates from 10 to 100 mV s^−1^. (**d**) GCD results for RH-900 at current densities from 0.5 to 10 A g^−1^. (**e**) CVs of Act-RH-900 at scan rates from 10 to 100 mV s^−1^. (**f**) GCD results for Act-RH-900 at current densities from 0.5 to 10 A g^−1^.

The electrochemical performances of the as-prepared carbon materials were measured by CV and GCD tests in a three-electrode system with 1 M KOH aqueous electrolyte (Figure 7). The cyclic voltammograms of the samples under various conditions obtained at a scan rate of 50 mV s^−1^ are displayed in Figure 7a. The voltammograms adopted a nearly rectangular shape without clear oxidation or reduction peaks, which indicated that the capacitance of the electrode was supplied almost entirely by the electrostatic double-layer conductor (EDLC) [47,48]. Additionally, the integrated area of the closed CV curve reflects the capacitance of electrode materials. The CVs of RH-900 and Act-RH-900 had the largest areas at the scan rate of 50 mV s^−1^, indicating that they obtained the maximum capacitance values. These results were attributed to their micro/mesoporous structures, reasonable pore distributions, and content of disordered graphitization structures [49]. To further explore the reason for the superior capacitor performance of RH-900 and Act-RH-900, the CV curves were obtained at various scan rates (10–100 mV s^−1^), and the results are shown in Figure 7c,e, respectively. The curve shapes remained generally stable as the scan rates increased, which confirmed the stable carbon structure of the samples [50]. Meanwhile, the integrated areas of the CV closed curves increased with the increasing scan rate, regardless of the voltage window. The most noticeable effect on the CV areas corresponded to a voltage window of –1 to 0 V for RH-900, and –1 to 0.4 V for Act-RH-900, which indicated that supercapacitor electrodes comprising RH-900 and Act-RH-900 could accommodate significant energy storage [51]. Longer discharge times were observed for RH-900 and Act-RH-900 (Figure 7b), thus demonstrating the superior capacitance properties of these two materials. As shown in Figure 7d,f, RH-900 and Act-RH-900 exhibited quasi-isosceles triangle shapes when the electrodes were charged to 0.3 V, and the longer discharge times corresponded to a current density of 0.5 A g^−1^. 

The specific capacitance values of RH-800, RH-900, and RH-1000 were 47.2, 97.3, and 34.9 F g^−1^, respectively, at a current density of 0.5 A g^−1^. It is clear that RH-900 had superior electrochemical properties owing to its effective pore size distribution [52]. Showing a similar trend, the specific capacitance of the Act-RH-800, Act-RH-900, and Act-RH-1000 samples was 89.4, 150.8, and 20.8 F g^−1^, respectively, at a current density of 0.5 A g^−1^. The outstanding electrochemical properties of Act-RH-900 were attributed to defect structures [53]. As the current density increased, the specific capacitance of RH-900 and Act-900 decreased because the high current density accelerated the transfer of charge (electrons) and reduced the storage of electrolyte ions [54]. Actually, many works of carbon materials have been explored in Table 3, and the obtained sample (Act-RH-900) in this work has certain advantages in capacitance value.

**Figure 8:**
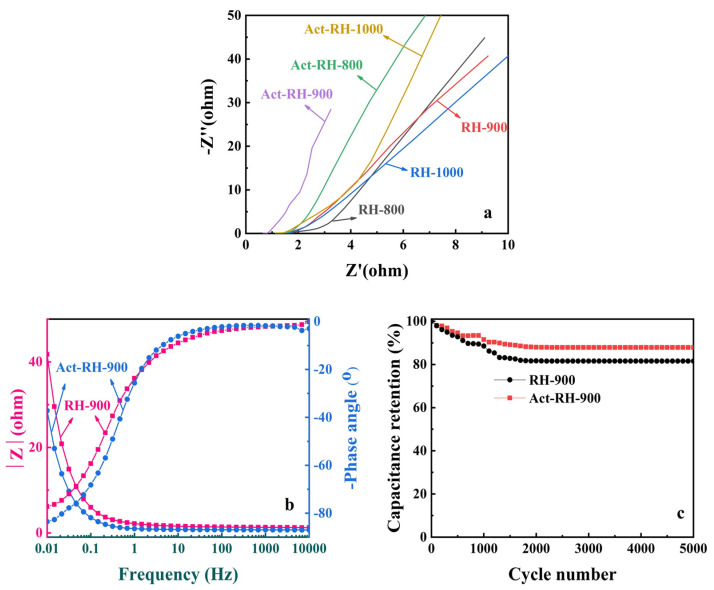
(**a**) EIS results for the investigated samples. (**b**) Bode phase angle and electrochemical impedance plots of RH-900 and Act-RH-900. (**c**) Capacitance retention of RH-900 and Act-RH-900 over 5000 cycles.

Representative Nyquist plots (from EIS measurements) for the samples are displayed in Figure 8a. The phase angle reached 45° in the low-frequency region, revealing the attendance of supercapacitor performance. In the high-frequency region, the Act-RH-900 curve exhibited a smaller slope, which indicated lower impedance. The extension line value crossing the horizontal axis represents the solution resistance (Rs), and the Rs of Act-RH-900 is smaller than the other samples, which shows that the working electrode made by Act-RH-900 suffers relatively low resistance in the energy-storage process. This is due to the fact that the unique pore structure can be adequately accessed by ions to reduce the resistance of the electrode material, and the silicon-containing functional groups can improve the conductivity of the materials. These results further highlighted the advantage of Act-RH-900 for application as an electrochemical energy-storage material [58]. The Bode phase angle plots of RH-900 and Act-RH-900 are presented in Figure 8b. The phase angles of RH-900 and Act-RH-900 in the low-frequency region were about –77° and –84°, respectively. These angles were between –90° (ideal capacitor) and –45° (pseudo-capacitor), which indicated intercalation capacitance in the RH-900 and Act-RH-900 materials [59]. The RH-900 and Act-RH-900 feature frequency corresponding to a phase angle of –45° is 0.23 and 0.38 Hz, respectively. The time constant of RH-900 and Act-RH-900 can reach to 4.35 and 2.63 s, respectively, which are much smaller than 10 s (the time constant of conventional activated carbon SCs). The smaller time constant demonstrated that there was a faster charge/discharge rate, and the rate of Act-RH-900 is smaller than that of RH-900. Meanwhile, RH-900 and Act-RH-900 achieved capacitance retentions greater than 81.56% and 87.88%, respectively, after 5000 cycles (Figure 8c). These results illustrate the excellent cycling stability and electrochemical reversibility of the Act-RH-900 sample in particular [60]. The pore size distribution and defect structure of the Act-RH-900 sample have important effects on the material’s electrochemical energy-storage properties: the micropores provide storage space, and mesopores provide channels for ion transport, while the defect structure enhances the ion contact area [61,62]. The synergism among the micro- and meso-porous structural characteristics enables efficient electrochemical transport and storage.

**Figure 9:**
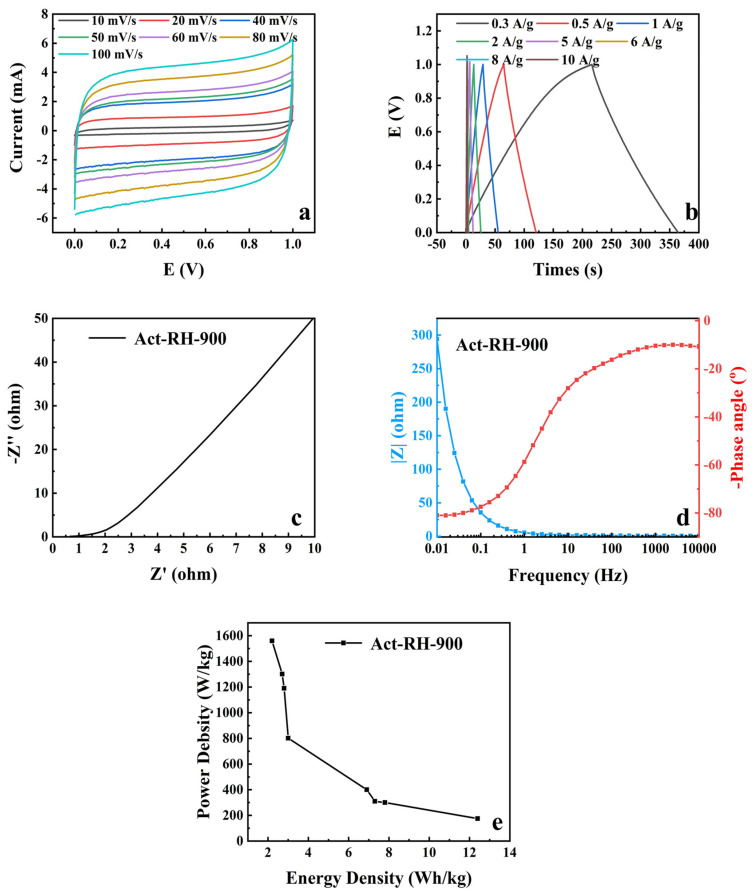
Electrochemical performances of Act-RH-900//Act-RH-90 tested in the two-electrode system. (**a**) CVs of Act-RH-900 at scan rates from 10 to 100 mV s^−1^. (**b**) GCD results for Act-RH-900 at current densities from 0.3 to 10 A g^−1^. (**c**) EIS results for Act-RH-900. (**d**) Bode phase angle and electrochemical impedance plots of Act-RH-900. (**e**) Ragone plot for Act-RH-900.

The symmetric SCs were further explored depending on the better capacitive performance of Act-RH-900 in the three-electrode system. The symmetric SCs (Act-RH-900//Act-RH-900) were tested in the two-electrode system in 1 M KOH aqueous electrolyte. Figure 9a shows the CV graph of Act-RH-900//Act-RH-900 working in the scan range of 10–100 mV s^−1^. The voltammograms adopted a nearly rectangular shape without clear oxidation or reduction peaks, which indicated that the capacitance of the electrode was supplied almost entirely by the electrostatic double-layer conductor (EDLC). The curve shapes remained generally stable as the scan rates increased, which confirmed that Act-RH-900//Act-RH-900 could withstand a wide voltage from 0 to 1 V, and also proved the stable structure of the sample carbon material. The highly symmetrical triangle shape for the GCD curves of Act-RH-900//Act-RH-900 at different current densities of 0.3, 0.5, 1, 2, 5, 6, 8, and 10 A g^−1^ is displayed in Figure 9b, which indicates that the sample shows ideal capacitive behavior because of the structure of the material. According to Equations (1) and (2), a single-electrode specific capacitance of 89.28 F g^−1^ at a current density of 0.3 A g^−1^ for Act-RH-900//Act-RH-900 has been calculated by discharging time. Act-RH-900//Act-RH-900 has obtained an excellent energy density of 12.4 Wh kg^−1^ and power density of 175 W kg^−1^ in the 1 M KOH electrolyte calculated by the Equations (3) and (4). A representative Nyquist plot (from EIS measurements) for the Act-RH-900//Act-RH-900 is displayed in Figure 9c and the Bode phase angle plot is presented in Figure 9d. The phase angle of Act-RH-900//Act-RH-900 in the low-frequency region was about –79°. The angle was between –90° (ideal capacitor) and –45° (pseudo-capacitor), which indicated intercalation capacitance in the Act-RH-900//Act-RH-900 materials. These results are similar to the three-electrode test results. The Ragone plot of Act-RH-900//Act-RH-900 is shown in Figure 9e. The power density decreased as the energy density increased.

## 4. Conclusions

Rice hull-derived porous Si-carbon materials were prepared via a simple and effective carbonization process from biomass waste (rice hull) without any loading or supplement addition. The structures and pore size distributions of the rice hull-based carbon materials were controlled effectively by tuning the carbonization and alkali activation mixing processes. Following alkali activation, the samples contained numerous pores and displayed coral reef-like structures surrounded by scale-like defects. The pore structures of the samples changed from mainly microporous to a combination of mesoporous and microporous morphologies as the carbonization temperature increased from 800 to 1000 °C. Among the investigated materials, Act-RH-900 had the highest surface area (768 cm^2^ g^−1^), largest pore volume (0.563 cm^3^ g^−1^), and optimal pore size ratio (S_micro_/S_BET_ = 83%). The presence of silicon influenced the formation of the pore structure, as well as the pore size distribution. For example, Si could change the pore structure from disordered to partially ordered, and increase the number of micropores. In general, micropores provided storage space and mesopores provided channels for ion transport, while defect structures enhanced the contact area with ions. The obtained micro/mesoporous character and structural features are beneficial for creating electrochemical devices on supercapacitors. Importantly, the Act-RH-900 exhibited a high specific capacitance (150.8 F g^−1^), high energy density (31.9 Wh kg^−1^), and high-power density (309.2 W kg^−1^) at a current density of 0.5 A g^−1^ in 1 M KOH electrolyte. This work provides a new route for achieving efficient valorization of silicon-fixing biomass waste, which expands the scope of silicon-fixing biomass utilization in the field of electrochemical energy storage.

## Figures and Tables

**Table 1:** Composition of RH-900 and Act-RH-900 samples (atomic ratios).

	Atom	C	O	Si	Others
Sample					
RH-900		73.23	17.53	6.7	2.54
Act-RH-900		68.26	20.79	8.12	2.83

**Table 2:** Pore structure parameters of the investigated samples.

Sample	S_BET_ (m² g^−1^)	S_micro_/S_BET_ (%)	Pore Volume (cm³ g^−1^)	Average Pore Size (nm)
RH-800	226	95	0.136	1.2
RH-900	418	93	0.246	1.1
RH-1000	286	77	0.224	1.7
Act-RH-800	732	80	0.541	1.4
Act-RH-900	768	83	0.563	1.4
Act-RH-1000	229	65	0.511	4.4

**Table 3:** The specific capacitance of Act-RH-900 and other materials.

Materials	Electrolyte	Current Density (A g^−^^1^)	Specific Capacitance (F g^−1^)	Ref.
Carbon fibers	Na_2_SO_4_	0.5	74	[55]
M-NMCCs-1073	KOH	1	128	[12]
Modified graphene	KOH	0.5	135	[23]
DPT-HPP	KOH	0.5	110	[30]
DAB-TFP COF	H_2_SO_4_	0.5	98	[56]
TpPa-COF@PANI	H_2_SO_4_	0.2	95	[57]
Act-RH-900	KOH	0.5	150.8	This work

## Data Availability

The data presented in this study are available on request from the corresponding author.
